# Statin use and its effect on all-cause mortality of melanoma patients: a population-based Dutch cohort study

**DOI:** 10.1002/cam4.285

**Published:** 2014-06-17

**Authors:** Elisabeth Livingstone, Loes M Hollestein, Myrthe P P van Herk-Sukel, Lonneke van de Poll-Franse, Arjen Joosse, Bastian Schilling, Tamar Nijsten, Dirk Schadendorf, Esther de Vries

**Affiliations:** 1 Department of Dermatology, University Hospital Duisburg-EssenEssen, Germany; 2 Department of Dermatology, Erasmus MC UniversityRotterdam, The Netherlands; 3 PHARMO Institute for Drug Outcome ResearchUtrecht, The Netherlands; 4 CoRPS - Center of Research on Psychology in Somatic Diseases, Tilburg UniversityThe Netherlands; 5 Comprehensive Cancer Centre South (CCCS), Eindhoven Cancer RegistryEindhoven, The Netherlands; 6 Department of Public Health, Erasmus MC UniversityRotterdam, The Netherlands

**Keywords:** HMG-CoA reductase inhibitor, melanoma, statin, survival

## Abstract

Preclinical data showed anticancer effects of statins in melanoma, but meta-analyses could not demonstrate a reduced melanoma incidence in statin users. Rather than preventing occurrence, statins might reduce growth and metastatic spread of melanomas and ultimately improve survival. In this population-based study, we investigated the relationship between statin use and survival of melanoma patients. Patients ≥18 years who were diagnosed with cutaneous melanoma (Breslow thickness >1 mm) and registered in the Eindhoven Cancer Registry and in PHARMO Database Network between 1 January 1998 and 31 December 2010 were eligible. The hazard ratio (HR) of all-cause mortality was calculated by employing adjusted time-dependent and time-fixed Cox proportional hazard models. Disease-specific survival was estimated by means of 3-year relative survival rates (RSR). A control cohort of randomly selected patients using statins from PHARMO Database Network matched on age and gender was used to compare RSR of statin users to the general population. After melanoma diagnosis, 171 of 709 patients used statins. Use of statins showed a nonsignificantly decreased hazard of death (adjusted HR 0.76, 95% confidence interval [CI] 0.50–1.61). After stratification for gender, male but not female statin users showed a favorable outcome compared to nonusers (HR 0.57, 95% CI 0.32–0.99; HR 1.22, 95% CI 0.62–2.38, respectively). Three-year RSR for male statin users tended to be higher than for nonusers (91% vs. 80.5%, *P* = 0.06), no differences were observed in women (87.1% vs. 92.5%, *P* = 0.76). Statin use was not associated with an improved survival of melanoma patients. The trend for better survival of male in contrast to female statin users warrants further research.

## Introduction

Melanoma is the deadliest form of skin cancer and responsible for 80% of skin cancer deaths 1. Results from a recent analysis predict a continuous melanoma incidence rise in Europe, especially in the Nordic and north-western European countries 2. Despite novel treatment options 3,4, melanoma—once metastasized beyond locoregional sites—is incurable in most patients.

Statins are frequently used to prevent cardiovascular diseases and block the enzyme 3-hydroxy-3-methylglutaryl-coenzyme A (HMG-CoA) reductase, which catalyzes the conversion of HMG-CoA to mevalonate, the rate-limiting step in de novo cholesterol synthesis 5. They may also have anticancer properties—either as a direct effect of the lowered cholesterol levels leading to decreased proliferation and migration of cancer cells 6,7, a reduction in the downstream products of the mevalonate pathway 8,9, or through other pleiotropic effects on the cellular level.

Particularly in melanoma, antiproliferative, proapoptotic, and immunomodulatory effects of statins have been shown in cell lines 10–14 and mouse models 14–17. Inhibition of the mevalonate metabolic pathway seems to be one of the major factors as it is essential for membrane formation and isoprenylation of several small GTPases involved in cell growth and differentiation. Among the GTPases requiring isoprenylation are the proteins of the Rho family including RhoA, Rac1, and Cdc42, which regulate signal transduction from receptors in the membrane in a variety of cellular events, thereby acting as molecular switches in the cell 18. Kidera et al. 15 could demonstrate a significantly decreased membrane localization of Rho proteins in simvastatin-treated melanoma cells compared to the control cells. Additionally, oral administration of statins to mice significantly inhibited lung metastasis 15. Previously, atorvastatin was shown to inhibit Rho activation in vitro and in vivo metastasis of melanomas overexpressing RhoC, but did not affect cell growth in vivo 19.

Large meta-analyses of randomized controlled trials, however, could not find a significant association between statin use and a lower melanoma incidence 20–22, but population-based studies showed that statin use was associated with a reduced Breslow thickness 23 and that advanced melanomas were slightly more common among nonstatin users 24.

Rather than preventing the formation of a primary melanoma, statins might therefore reduce disease progression and/or melanoma-specific death as has been seen in other cancers 25–27. We decided to investigate the effect of statins on overall survival in melanoma patients in a large population-based cohort study in the Netherlands using data from the Eindhoven Cancer Registry (ECR) and the PHARMO Database Network hypothesizing that statin use after melanoma diagnosis would result in improved survival.

## Patients and Methods

### Study population and data collection

Data were retrieved from the linkage between the ECR and the PHARMO Database Network assuring high-quality and extensive information on statin exposure and melanoma diagnosis 28. The ECR is a population-based cancer registry in the South of the Netherlands covering 2.4 million inhabitants and includes more than 95% of all newly diagnosed malignancies 29. The registry is notified by six pathology departments, ten community hospitals (at 17 locations), and two large radiotherapy departments. Trained registration clerks actively collect data on diagnosis, patient characteristics, comorbidity, staging, and detailed information about initial treatment from hospital medical records. Linkage with the Dutch municipal records provided vital status until 31 December 2010.

PHARMO Database Network covers a demographic region of three million inhabitants and is a network of patient databases 28. The central patient database is linked to many databases 30. Relevant databases for this study include virtual complete longitudinal data obtained from community pharmacies (outpatient), hospital discharge records (Dutch National Medical Registration: LMR), a mortality registration, and a growing number of clinical laboratories, in-hospital pharmacies (inpatient), and general practitioners (these last three databases are available for a subcohort of the patients included in the PHARMO Database Network) 28. All pharmacy dispensed healthcare products on the Dutch market prescribed by medical practitioners are included in the community pharmacy database. Drug-dispensing records in PHARMO Database Network are virtually complete with regard to prescription drugs as previous studies demonstrated that most Dutch patients only visit one pharmacy 31,32.

A total of one million inhabitants are captured in the overlapping PHARMO-ECR catchment area. Follow-up of patients was either until patients moved away from the PHARMO-ECR catchment area, end of data collection of the specific community pharmacy, or end of study or death, whichever occurred first.

### Study population

**Figure 1:**
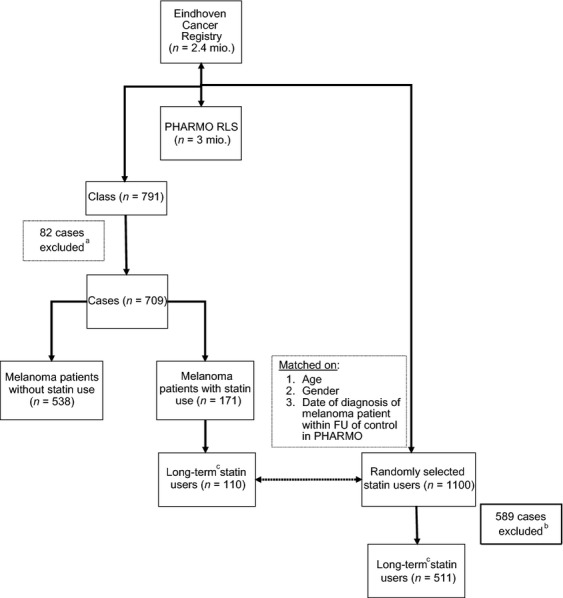
Study population selection and matching. ^a^Eighty-two cases excluded as <1 year FU in PHARMO Database Network. ^b^Five hundred forty-six randomly selected statin users excluded as no long-term statin users, 43 cases excluded as <1 year FU in PHARMO Database Network. ^C^statin (≥2) dispensings in the year prior to and statin use at the time of melanoma diagnosis. ECR, Eindhoven Cancer Registry; FU, follow-up.

Patients (≥18 years) registered in the ECR with a diagnosis of invasive cutaneous melanoma with a tumor thickness >1 mm between 1 January 1998 and 31 December 2010 who were also registered in PHARMO Database Network at the time of melanoma diagnosis were eligible (*N* = 791) (Fig.1).

A control cohort of 1100 randomly selected statin users of the PHARMO Database Network cohort matched on age, gender, and index date in a 1:10 ratio to the statin-using melanoma patients was constructed to compare the survival of statin users to the general population. The date of melanoma diagnosis was used as the index date of the matched controls (this required that the date of melanoma diagnosis of the melanoma patient had to be within the follow-up time in PHARMO Database Network of the matched control). The central bureau for genealogy, the local pharmacy, or the hospital served as sources for the date of death of the matched statin users.

A minimum of 1 year of follow-up in the PHARMO Database Network was required for patients and controls prior to melanoma diagnosis or index date, respectively, to determine potential confounders and drug exposure in the year prior to diagnosis, leaving 709 melanoma patients and 511 control cases for final analyses. The date of melanoma diagnosis (melanoma cohort) and the index date (matched cohort) corresponded with the start of follow-up.

### Statin use

Information on dispensing including anatomical therapeutical chemical (ATC) code, date dispensed, and days' supply was obtained from the PHARMO Database Network. Dispensings with the ATC code group C10AA (HMG-CoA reductase inhibitors), C10BA (HMG-CoA reductase inhibitors in combination with other lipid modifying agents) and C10BX (HMG-CoA reductase inhibitors, other combinations) of the WHO Collaborating Centre for Drug Statistics Methodology (Table 1) were considered statin dispensings. For combination drugs, only the statin was considered.

**Table 1:** Frequency of all dispensed statins in statin patients after melanoma diagnosis (80.4% lipophilic)

Drug name^1^	Lipophilic	Hydrophilic	Number of dispensings	Percentage of total
Atorvastatin	x		1361	28.7
Cerivastatin^2^	x		16	0.3
Fluvastatin	x		187	3.9
Pravastatin		x	575	12.1
Rosuvastatin		x	356	7.5
Simvastatin	x		2244	47.4
Total			4739	100

1 Lovastatin, a commonly prescribed statin in many countries, is not on the market in the Netherlands.

2 Cerivastatin has been withdrawn from clinical use in 2001.

By dividing the amount of dispensed drug by the number of pills prescribed per day as defined in the pharmacy data, the duration of each dispense was calculated. The number of overlapping days was added to the dispensing duration of the second dispensing, if two dispensings overlapped. To be able to compare the dosage of different types of statins, the defined daily dose system (DDD) of the WHO 33 was used. For each individual patient, the DDD equivalent was calculated by multiplying the amount of pills dispensed by the corresponding dosage per pill and divided by the DDD.

### Other covariates

The following variables were considered potential confounders: age at diagnosis, gender, histological subtype, location of the primary melanoma, tumor thickness (categorical variable), nodal and distant metastases, and comorbidities (any and specific comorbidities). As a proxy of general morbidity as well as a proxy of healthcare- and pharmacy-seeking behavior, the number of distinct medication classes dispensed (unique ATC codes) excluding statins and unique hospital admissions in the year prior to diagnosis were also considered potential confounders (both as continuous variables). All variables that were considered potential confounders were also considered potential effect modifiers and tested for interaction.

### Statistical analysis

To compare relevant characteristics of users and nonusers, χ^2−^ tests and Fisher's exact tests were used for categorical variables and Student's *t*-test for continuous variables. Cox proportional hazard (PH) models were used to analyze the association between statin use and survival. Time since diagnosis was the underlying timescale.

Statin use before diagnosis was defined as having at least two statin dispensings during the year prior to melanoma diagnosis and use of statins at the time of diagnosis. A time-varying covariate was used for statin use after diagnosis, where patients were considered statin users since first statin use after diagnosis. Duration and dose of statin use after diagnosis were also taken into account as time-varying covariates. In these analyses, the number of cumulative days of statin use of the subject with the event of interest is compared with the cumulative statin use of all other subjects at the same time point. Covariates which influenced the age and sex-adjusted HR by more than 10% were considered potential confounders and added in the multivariable analysis.

As information on cause of death was not accessible, we calculated 3-year relative survival rates (RSRs) as a proxy for disease-specific mortality. The RSR is the absolute survival rate divided by the expected survival rate in the period of diagnosis from the general population with the same sex and age structure 34,35. By calculating RSR for the statin-using and nonusing melanoma patient groups, an estimate of melanoma-specific survival is being made. Such an analysis is a good and valid alternative if cause-specific death is not available and can sometimes even be more accurate than estimating cause-specific survival by using death certificates because the primary cause of death is often not clear and inter-doctor variation in the cause of death ascertainment is common. As immortal time bias 36 might occur when statin use after diagnosis were investigated in the RSR calculations, statin use before diagnosis was assessed using the aforementioned definition. Graphs for time interval-specific hazard ratios (HRs) were visually inspected and showed no violation of the PH assumption. All statistical tests were two sided with a rejection of the null hypothesis at *P* < 0.05. Analyses were performed using SPSS 20.0 (IBM Corp., Armonk, NY) and SAS 9.3 (SAS Institute Inc., Cary, NC).

## Results

### Study population

The baseline characteristics are shown in Table 2 (refer to Table S1 for characteristics stratified on gender). In total, 171 (24.1%) of the 709 eligible melanoma patients used statins after melanoma diagnosis. During 2632 patient years of follow-up, 159 patients died. Patients who used statins were more likely to be male (58.5%), older (67.3 vs. 58.0 years), and to have a longer follow-up time (3.5 years vs. 2.9 years) than nonusers. There was no significant difference for tumor thickness or nodal or distant metastasis status between groups (Table 2). Comorbidities, hospitalizations, and medication use were more prevalent in the statin user group as expected. The higher rate of distant metastases of female statin users at initial diagnosis (4.2%) compared to males (0% for statin users and 2.6% for nonusers) and to female nonusers (1.9%) was statistically nonsignificant and is possibly influenced by the low numbers of patients with distant metastases in general. Female patients with statin use have a higher rate of other cancers compared to males and to female nonusers.

**Table 2:** Patient and tumor characteristics

Characteristics	Statin users (*N* = 171)^1^	Nonusers (*N* = 538)	*P*
Gender, *N* (%)			
Male	100 (58.5)	268 (49.8)	0.05
Female	71 (41.5)	270 (50.2)	
Age^2^ (year)			
Mean (SD)	67.3 (11.7)	58.0 (16.5)	<0.001
Median (IQR)	70 (60–77)	59 (46–70)	
Time of FU			
Years, median (IQR)	3.5 (1.6–5.8)	2.9 (1.3–5.3)	0.02
Number of deaths *N* (%)	40 (23.4)	119 (22.1)	0.73
Histological subtype, *N* (%)			
SSM	91 (53.2)	257 (47.8)	0.09
NMM	30 (17.5)	147 (27.3)	
LMM	4 (2.3)	8 (1.5)	
ALM	2 (1.2)	5 (0.9)	
Others	44 (25.7)	121 (22.5)	
Body site of the melanoma, *N* (%)			
Head and neck	30 (17.5)	81 (15.1)	0.36
Trunk	65 (38.0)	191 (35.5)	
Upper extremity	37 (21.6)	106 (19.7)	
Lower extremity	39 (22.8)	160 (29.7)	
Tumor thickness, *N* (%)			
≥1.01 and ≤2	83 (48.5)	287 (53.3)	0.39
≥2.01 and ≤	60 (35.1)	159 (29.6)	
≥4.01	28 (16.4)	92 (17.1)	
Nodal metastases,^2^ *N* (%)	26 (15.2)	78 (14.5)	0.82
Distant metastases,^2^ *N* (%)	3 (1.8)	12 (2.2)	1.00
Comorbidities,^2^ *N* (%)			
Any	105 (61.4)	162 (30.1)	<0.001
Hypertension	46 (26.9)	69 (12.8)	<0.001
Heart diseases	58 (33.9)	39 (7.2)	<0.001
Cancer	32 (18.7)	57 (10.6)	0.01
Stroke	11 (6.4)	7 (1.3)	<0.001
Diabetes	22 (12.9)	20 (3.7)	<0.001
Lung diseases	10 (5.8)	24 (4.5)	0.22
Gastrointestinal diseases	8 (4.7)	7 (1.3)	0.01
Unique hospitalizations,^3^ *N* (%)			
No admissions	130 (76.0)	464 (86.2)	0.01
1 admission	31 (18.1)	55 (10.2)	
>1 admission	10 (5.8)	19 (3.5)	
Unique ATC codes,^3^ *N* (%)			
0 ATC codes	11 (6.4)	100 (18.6)	<0.001
1–3 ATC codes	36 (21.4)	240 (44.6)	
>3 ATC codes	124 (72.5)	198 (36.8)	
Average DDD, mean (SD)	0.97 (0.55)	n.a.	n.a.
Average statin exposure in days, mean (SD)	959.8 (882.0)	n.a.	n.a.

ATC, anatomical therapeutic chemical classification system; FU, follow-up; IQR, interquartile range; *N*, total number of patients.

1 Statin user after melanoma diagnosis.

2 At the time of initial melanoma diagnosis.

3 In the year prior to diagnosis.

Patient and tumor characteristics of the cohort with statin use before melanoma diagnosis were very similar and presented in Table S2.

### Statin use and HRs of all-cause death

**Figure 2:**
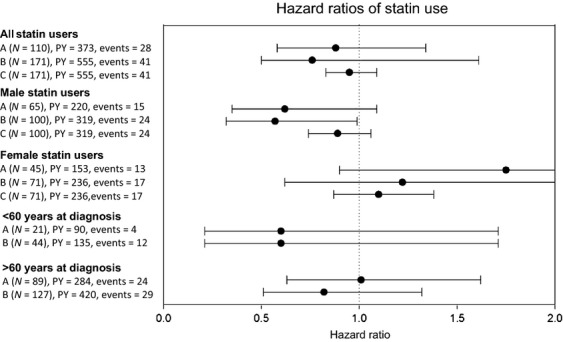
Hazard of death and 95% confidence interval for melanoma patients with statin use compared to nonusers. A = statin use before melanoma diagnosis. B = statin use after melanoma diagnosis. C = per additional year of use for statin users after melanoma diagnosis. PY, person years.

Almost half of all dispensings were for simvastatin (47.4%), followed by atorvastatin (28.7%); 80.4% were lipophilic (Table 1). Statin use before or after diagnosis was not significantly associated with a reduction in the hazard of death (adjusted HR before diagnosis 0.88, 95% confidence interval [CI] 0.58–1.34; adjusted HR after diagnosis 0.76, 95% CI 0.50–1.61) (Fig.2, Table S3).

Duration of statin exposure was also not associated with all-cause mortality (adjusted HR per additional year of statin use 0.95, 95% CI 0.83–1.09). Neither the amount of average or cumulative DDDs nor a combination of duration and dosage changed the HR significantly (Table S4).

Statistical significant interaction was observed between statin use and age, gender, unique ATC, and gastrointestinal diseases. Due to low patient numbers, stratification on unique ATC and gastrointestinal diseases was not possible and other stratified analyses should therefore be considered as exploratory. Overall, males have a worse prognosis than females (HR 1.74, 95% 1.25–2.43). Stratification for gender showed that male statin users have a favorable HR of death (statin use before melanoma: HR 0.62, 95% CI 0.35–1.09, after melanoma diagnosis: HR 0.57, 95% CI 0.32–0.99) compared to male nonusers. In women, however, there was no effect of statin use (statin use before melanoma: HR 1.75, 95% CI 0.90–3.38, after melanoma diagnosis: HR 1.22, 95% CI 0.62–2.38). Further stratification according to duration of exposure and dosage yielded similar results, although all statistically nonsignificant (Table S4). Stratification on nodal metastases (yes vs. no) at the time of diagnosis did not show a significant impact of statin use depending on nodal stage (data not shown).

### Relative survival analyses

**Figure 3:**
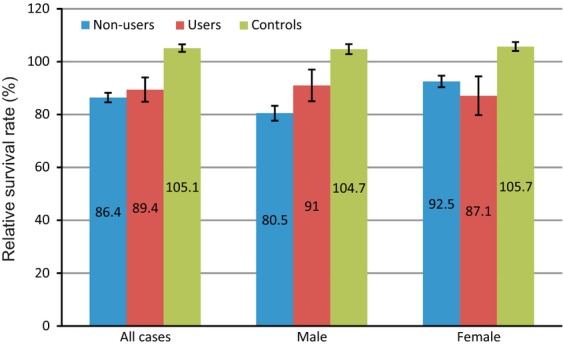
Three-year relative survival rate and standard error of the melanoma patients and controls. The relative survival rate of all melanoma patients and female melanoma patients with statin use was comparable to the nonusers, whereas male statin users may have a 3-year RSR superior to the nonusers, although not statistically significant (91.0% vs. 80.5%, *P* = 0.06).

The 3-year crude survival of melanoma patients of nonusers and statin users was comparable (80.5% vs. 78.8%, *P* = 0.27, Table 3). The 3-year RSR was also comparable (86.4% vs. 89.4%, *P* = 0.27, Fig.3). The 3-year RSR of randomly selected statin users was better than that of the general population (105.1%; 95% CI 102.5–107.8).

**Table 3:** Three-year crude and relative survival rates for statin users, nonusers, and the matched control cohort

	*N*	Events	Three-year crude survival KM (%)	95% CI	*P*^1^	Three-year relative survival (%)	95% CI	*P* 1
Melanoma patients^2^								
Nonuser	599	131 (21.9%)	80.5	77.0–84.0	Referent	86.4	82.6–90.2	Referent
Male	303	90 (29.7%)	74.2	68.7–79.7	Referent	80.5	74.7–86.4	Referent
Female	296	40 (13.5%)	87.3	83.0–91.6	Referent	92.5	87.9–97.0	Referent
Statin user	110	28 (25.5%)	78.8	70.2–87.4	0.27	89.4	80.3–98.5	0.27
Male	65	15 (23.1%)	79.1	67.9–90.3	0.44	91.0	79.3–102.8	0.06
Female	45	13 (28.9%)	78.3	64.8–91.8	0.006	87.1	72.9–101.3	0.76
Matched control cohort^3^								
Statin user	511	42 (8.2%)	94.2	91.8–96.6	n.a.	105.1	102.5–107.8	n.a.
Male	309	30 (9.7%)	93.0	89.6–96.4	n.a.	104.7	100.9–108.5	n.a.
Female	202	12 (5.9%)	96.1	93.0–99.2	n.a.	105.7	102.3–109.1	n.a.

CI, confidence interval; n.a., not applicable.

1 Log-rank test for crude survival, *z*-test for proportions for relative survival.

2 Chronic statin users. Minimum 2 dispensings for statin within 1 year prior to melanoma diagnosis and use at time of melanoma diagnosis.

3 Control cohort of statin users without melanoma diagnosis matched on age and gender.

Stratification for gender showed that male statin users may have a 3-year RSR superior to the nonusers, although not statistically significant (91.0% vs. 80.5%, *P* = 0.06), whereas no difference of 3-year RSR for female patients was seen (87.1% vs. 92.5%, *P* = 0.76). These gender differences were not found in the randomly selected statin users, where 3-year RSR of males and females were comparable (Table 3).

## Discussion

In our cohort of 709 melanoma patients with a Breslow thickness >1 mm, statin use did not significantly impact overall survival, but our results indicate that statin use might lead to differential survival of melanoma patients depending on gender. Neither timing nor duration or dosage of statin use changed the hazard of death significantly. Stratification on gender, however, demonstrated possible superior survival of statin users compared to nonusers in males only.

The currently proposed potential anticancer effects of statins include the induction of melanoma cell apoptosis, the inhibition of proliferation and invasion, the prevention of the activation of key proteins for cell cycle regulation, and the stimulation of antimelanoma immune responses 10–14,16,17,37–42.

Only one other population-based study investigated statin use and cancer mortality in melanoma among several other cancer types 43. Danish patients using statins were 15% less likely to die from any cause (HR 0.85, 95% CI 0.83–0.87) and from cancer (HR 0.85, 95% CI 0.82–0.87). A reduced cancer-related mortality for statin users was found in 13 different cancers including lung, colorectal, prostate and breast, but not in melanoma (HR 1.21, 95% CI 0.95–1.52). A subanalysis for gender in melanoma patients was not performed, but the HR for all cancer patients and cancer-related mortality showed that the HR of death in male patients using statins was reduced more than in female patients (HR 0.82, 95% CI 0.81–0.86 vs. HR 0.92, 95% CI 0.88–0.92).

The female survival advantage in melanoma in general was confirmed in our study. The favorable results of statin use only in male statin users are therefore surprising. Cancer survival has been shown to be generally better in females than in males for most cancers 44, especially in melanoma 45,46. Even after adjustment for potential behavioral differences (primarily diagnostic delay and healthcare consumption), sex remains an independent prognostic factor for melanoma progression and survival 45. Biological differences are therefore highly likely to play a role.

When investigating the 3-year RSR differences across gender in our study group more closely (Fig.2, Table S2), male nonusers have a poor prognosis (80.5%) compared to either female nonusers (92.5%) or statin users (89.4%). However, statin use in males improves their prognosis to levels comparable to female patients (91.0%). Statin use somehow seems to negate the male survival disadvantage in melanoma. Therefore the effects of statins on melanoma might be related to the underlying mechanism of the overall gender differences in melanoma survival. We suggest two mechanisms influenced by statins which might be related to the overall male disadvantage in melanoma survival.

First, Krauthammer et al. 47 reported that somatic activating Rac1 mutations, ranking third after BRAF- and NRAS-mutations, in general, occur significantly more often in men than in women. As statins have been shown to prevent Rac1 isoprenylation 15 and to inhibit the Rho-pathway 15,19, it might be possible that males have worse survival rates than females due to a higher rate of Rac1 mutations leading to an increased activity of the Rho-pathway in male melanoma cells, which in turn might be counteracted by statin use. Second, as melanoma is a highly immunogenic tumor 48 and males have a weaker immune system than females 49, it might be possible that males benefit more from the activating effect of statins on the antimelanoma immune response 14,17,42 than females, explaining the differential effect of statin use across gender. However, the noticed gender differences might also only be an epiphenomena with the underlying cause being associated with gender but not caused by gender.

The most noteworthy strengths of this study are its prospective nature and the validated data from two large, nationally representative and linked cancer- and pharmacy databases, which provided detailed information on patient demographics, patient outcomes, tumor characteristics and, importantly, dose, duration, and timing of statin exposure.

There are some limitations that need to be addressed. As the cause of death was unknown, only all-cause rather than cancer-specific mortality could be assessed. We therefore calculated RSR as a proxy for disease-specific survival. With the inclusion of randomly selected statin users we demonstrated that relative survival of statin users is better than the general age- and gender-matched population. This implies that part of the improved 3-year survival rate in male melanoma patients with statin use may also be attributed to a decreased death risk due to cardiovascular comorbidities.

We had insufficient data to investigate separately for lipophilic and hydrophilic statins. Some studies, especially in breast cancer could only see a positive effect of lipophilic statins on in vivo breast cancer recurrence 25,26 and in vitro breast cancer growth 50. Only lipophilic statins are able to permeate the cell membrane and thus to directly affect cell proliferation, survival, and motility 51, hydrophilic statins require active carrier-mediated uptake, which is only present in hepatocytes. However, the majority of prescriptions in our cohort were for lipophilic statins (80.3%), it can thus be assumed that the results are primarily attributable to lipophilic rather than hydrophilic statins.

We had information on important possible confounders, such as cancer stage, comorbidities, and concomitant drug use. Other possible confounders, such as social and lifestyle factors (e.g., smoking) were not available through PHARMO Database Network and could therefore not be adjusted for. As the study was nonrandomized, residual confounding cannot be excluded.

In conclusion, our data did not show a significant beneficial effect of statins on survival of melanoma patients. The differential impact that statin use seems to have on male and female melanoma patients and possibly also other tumor entities requires further research in even larger cohorts and should include measures on cancer-specific outcomes. Additionally, when in vitro experiments are conducted, both male and female melanoma cell lines should be used to see if gender differences can be noticed. Future studies should also try to address effects of lipophilic versus hydrophilic statins.

## Supporting Information

**Table S1.** Patient and tumor characteristics stratified on gender for statin users after melanoma diagnosis versus nonusers. **Table S2.** Patient and tumor characteristics of the sensitivity analysis (statin use before melanoma diagnosis). **Table S3.** Cox proportional hazard ratios (HRs) for relevant prognostic factors for all-cause mortality for all melanoma patients and HR for statin use. **Table S4.** Cox proportional hazard ratios (HRs) for all-cause mortality for duration and dose of statin use after melanoma diagnosis.
