# Supplementary material for: Statin use and its effect on all-cause mortality of melanoma patients: a population-based Dutch cohort study
Source: Cancer Med. 2014 Jun 17;3(5):1284–93. doi: 10.1002/cam4.285 (PMC4302678; doi:10.1002/cam4.285)
Supplement: Supplementary file 1 [file cam40003-1284-SD1.docx]

**Table S1 - Supplemental table (online only)**

Patient and tumour characteristics stratified on gender for statin users after melanoma diagnosis vs. non-users.

|  | **MALE** | | | **FEMALE** | | |
| --- | --- | --- | --- | --- | --- | --- |
| **Characteristics** | **statin users (*N*=100)^a^** | **non-users (*N* =268)^b^** | ***p*^c^** | **statin users (*N*=71)^a^** | **non-users (*N* =270)^b^** | ***p*^c^** |
| Age^d^ -yr |  |  |  |  |  |  |
| mean (SD) | 66.0 (11.7) | 57.6 (15.3) | <0.001 | 69.9 (11.5) | 58.4 (17.5) | <0.001 |
| Time of FU |  |  |  |  |  |  |
| Years, median (IQR) | 3.5 (1.6-6.2) | 2.6 (1.2-4.8) | 0.007 | 3.4 (1.4-5.7) | 3.7 (1.5-5.6) | 0.62 |
| Number of deaths - N (%) | 24 (24.0) | 81 (30.2) | 0.24 | 16 (22.5) | 30 (14.1) | 0.08 |
| Histological subtype - no. (%) |  |  | 0.3 |  |  | 0.21 |
| SSM | 52 (52.0) | 134 (50.0) |  | 39 (54.9) | 123 (45.6) |  |
| NMM | 20 (20.0) | 76 (28.4) |  | 10 (14.1) | 71 (26.3) |  |
| LMM | 2 (2.0) | 2 (0.7) |  | 2 (2.8) | 6 (2.2) |  |
| ALM | 2 (2.0) | 3 (1.1) |  | 0 (0.0) | 2 (0.7) |  |
| Others | 24 (24.0) | 53 (19.8) |  | 20 (28.2) | 68 (25.2) |  |
| Body site of the melanoma - N (%) |  |  | 0.95 |  |  | 0.37 |
| Head and neck | 19 (19.0) | 43 (16.0) |  | 11 (15.5) | 38 (14.1) |  |
| Trunk | 49 (49.0) | 125 (46.6) |  | 16 (22.5) | 66 (24.4) |  |
| Upper extremity | 19 (19.0) | 44 (16.4) |  | 18 (25.4) | 62 (23.0) |  |
| Lower extremity | 13 (13.0) | 56 (20.9) |  | 26 (36.6) | 104 (38.5) |  |
| Tumour thickness - N (%) |  |  | 0.44 |  |  | 0.89 |
| >=1,01 and <= 2 | 43 (43.0) | 130 (48.5) |  | 40 (56.3) | 157 (58.1) |  |
| >=2,01 and <=4 | 38 (38.0) | 83 (31.0) |  | 22 (31.0) | 76 (28.1) |  |
| >=4,01 | 19 (19.0) | 55 (20.5) |  | 9 (12.7) | 37 (13.7) |  |
| Nodal metastases^f^ - N (%) | 15 (15.0) | 43 (16.0) | 0.81 | 11 (15.5) | 35 (13.0) | 0.58 |
| Distant metastases^f^  - N (%) | 0 (0.0) | 7 (2.6) | 0.2 | 3 (4.2) | 5 (1.9) | 0.37 |
| Comorbidities^f^  - N (%) | 58 (58.0) | 86 (23.1) | <0.001 | 47 (66.2) | 76 (28.1) | <0.001 |
| Any |  |  |  |  |  |  |
| Hypertension | 25 (25.0) | 28 (10.4) | 0.001 | 21 (29.6) | 41 (15.2) | 0.02 |
| Heart diseases | 35 (35.0) | 21 (7.8) | <0.001 | 23 (32.4) | 18 (6.7) | <0.001 |
| Cancer | 12 (12.0) | 39 (14.6) | 0.12 | 20 (28.2) | 18 (6.7) | <0.001 |
| Stroke | 5 (5.0) | 2 (0.7) | 0.01 | 6 (8.5) | 5 (1.9) | 0.03 |
| Diabetes | 13 (13.0) | 8 (3.0) | 0.001 | 9 (12.7) | 12 (4.4) | 0.51 |
| Lung diseases | 7 (7.0) | 14 (5.2) | 0.22 | 3 (4.2) | 10 (3.7) | 0.8 |
| Gastrointestinal diseases | 4 (4.0) | 4 (1.5) | 0.1 | 4 (5.6) | 3 (1.1) | 0.07 |
| Unique hospitalizations^g^ - no. (%) |  |  | 0.02 |  |  | 0.18 |
| no admissions | 74 (74.0) | 230 (85.8) |  | 56 (78.9) | 234 (86.7) |  |
| 1 admission | 20 (20.0) | 26 (9.7) |  | 11 (15.5) | 29 (10.7) |  |
| >1 admission | 6 (6.0) | 12 (4.5) |  | 4 (5.6) | 7 (2.6) |  |
| Unique ATC codes^g^ - N (%) |  |  | <0.001 |  |  | <0.001 |
| 0 ATC codes | 8 (8.0) | 61 (22.8) |  | 3 (4.2) | 39 (14.4) |  |
| 1-3 ATC codes | 24 (24.0) | 124 (46.3) |  | 12 (16.9) | 116 (43.0) |  |
| >3 ATC codes | 68 (68.0) | 83 (31.0) |  | 56 (78.9) | 115 (42.6) |  |
| Average DDD (mean, SD) | 0.98 (0.60) | n.a. | n.a. | 0.97 (0.48) | n.a. | n.a. |
| Average statin exposure in days (mean, SD) | 1004.3 (908.2) | n.a. | n.a. | 900.0 (847.8) | n.a. | n.a. |

Abbreviations

ATC, Anatomical Therapeutic Chemical classification system; FU, follow-up; IQR, interquartile range; *N*, total number of patients.

^a^Statin user after melanoma diagnosis.

^b^At the time of initial melanoma diagnosis.

^c^In the year prior to diagnosis.

**Table S2- Supplemental table (online only)**

Patient and tumour characteristics of the sensitivity analysis (statin use before melanoma diagnosis).

| **Characteristics** | **chronic statin user before MM (*N*=110)^a^** | **non-users (*N* =599)^b^** | ***p*** |
| --- | --- | --- | --- |
| Gender - N (%) |  |  |  |
| Male | 65 (59.1) | 303 (50.6) | 0.10 |
| Female | 45 (40.9) | 296 (49.4) |  |
| Age^d^ -yr |  |  |  |
| mean (SD) | 69.7 (10.3) | 58.6 (16.2) | <0.001 |
| median (IQR) | 71 (63-78) | 60 (47-71) |  |
| Time of FU |  |  |  |
| Years, median (IQR) | 3.1 (1.2-4.4) | 3.2 (1.4-5.6) | 0.18 |
| Number of deaths - N (%) | 28 (25.5) | 131 (21.9) | 0.41 |
| Histological subtype - no. (%) |  |  |  |
| SSM | 51 (46.4) | 297 (46.9) | 0.21 |
| NMM | 24 (21.8) | 153 (25.5) |  |
| LMM | 4 (3.6) | 8 (1.3) |  |
| ALM | 2 (1.8) | 5 (0.8) |  |
| Others | 29 (26.4) | 136 (22.7) |  |
| Body site of the melanoma - N (%) |  |  |  |
| Head and neck | 23 (20.9) | 88 (14.7) | 0.37 |
| Trunk | 40 (36.4) | 216 (36.1) |  |
| Upper extremity | 20 (18.2) | 123 (20.5) |  |
| Lower extremity | 27 (24.5) | 172 (28.7) |  |
| Tumour thickness - N (%) |  |  |  |
| >=1,01 and <= 2 | 56 (50.9) | 314 (52.4) | 0.42 |
| >=2,01 and <=4 | 39 (35.5) | 180 (30.1) |  |
| >=4,01 | 15 (13.6) | 105 (17.5) |  |
| Nodal metastases^c^ - N (%) | 18 (16.4) | 86 (14.4) | 0.59 |
| Distant metastases^c^ - N (%) | 3 (2.7) | 12 (2.0) | 0.72 |
| Comorbidities^c^ - N (%) |  |  |  |
| Any | 76 (69.1) | 191 (31.9) | <0.001 |
| Hypertension | 38 (34.5) | 77 (12.9) | <0.001 |
| Heart diseases | 44 (40.0) | 53 (8.8) | <0.001 |
| Cancer | 23 (20.9) | 66 (11.0) | 0.004 |
| Stroke | 9 (8.2) | 9 (1.5) | <0.001 |
| Diabetes | 12 (10.9) | 30 (5.0) | 0.01 |
| Lung diseases | 6 (5.5) | 28 (4.7) | 0.08 |
| Gastrointestinal diseases | 4 (3.6) | 11 (1.8) | 0.03 |
| Unique hospitalizations^d^ - no. (%) |  |  |  |
| no admissions | 81 (73.6) | 513 (85.6) | 0.01 |
| 1 admission | 22 (20.0) | 64 (10.7) |  |
| >1 admission | 7 (6.4) | 22 (3.7) |  |
| Unique ATC codes^d^ - N (%) |  |  |  |
| 0 ATC codes | 2 (1.8) | 109 (18.2) | <0.001 |
| 1-3 ATC codes | 20 (18.2) | 256 (42.7) |  |
| >3 ATC codes | 88 (80.0) | 234 (39.1) |  |
| Average DDD (mean, SD) | 0.92 (0.58) | n.a. | n.a. |

Abbreviations

ATC, Anatomical Therapeutic Chemical classification system; FU, follow-up; IQR, interquartile range; N, total number of patients.

^a^Min. 2 dispensings for statin within 1 year prior to melanoma diagnosis and use at time of melanoma diagnosis.

^b^No dispensing for statin or only short term use.

^c^At the time of initial melanoma diagnosis.

^d^In the year prior to diagnosis.

**Table S3- Supplemental table (online only)**

Cox proportional hazard ratios (HR) for relevant prognostic factors for all-cause mortality for all melanoma patients and HR for statin use.

|  | **Person years** | **Events** | **Multivariate HR^c^** | **95% CI** |
| --- | --- | --- | --- | --- |
| **Statin users** |  |  |  |  |
| statin use before melanoma diagnosis^a^ |  |  |  |  |
| Non-user (N=599) | 2261 | 131 | 1 |  |
| User (N=110) | 373 | 28 | 0.88 | 0.58-1.34 |
| statin use after melanoma diagnosis (*N*=171)^b^ |  |  |  |  |
| Non-user (N=538) | 2077 | 118 | 1 |  |
| User (N=171) | 555 | 41 | 0.76 | 0.50-1.61 |
| per additional year of use (N=171) | 555 | 41 | 0.95 | 0.83-1.09 |
| **Male statin users** |  |  |  |  |
| statin use before melanoma diagnosis (*N*=65)^a^ | 220 | 15 | 0.62 | 0.35-1.09 |
| statin use after melanoma diagnosis (*N*=100)^b^ | 319 | 24 | 0.57 | 0.32-0.99 |
| per additional year of use | 319 | 24 | 0.89 | 0.74-1.06 |
| **Female statin users** |  |  |  |  |
| statin use before melanoma diagnosis (*N*=45)^a^ | 153 | 13 | 1.75 | 0.90-3.38 |
| statin use after melanoma diagnosis (*N*=71)^b^ | 236 | 17 | 1.22 | 0.62-2.38 |
| per additional year of use | 236 | 17 | 1.10 | 0.87-1.38 |
| **Statin users ≤60 yrs at diagnosis** |  |  |  |  |
| statin use before melanoma diagnosis (*N*=21)^a^ | 90 | 4 | 0.60 | 0.21-1.71 |
| statin use after melanoma diagnosis (*N*=44)^b^ | 135 | 12 | 0.60 | 0.21-1.71 |
| per additional year of use | 135 | 12 | 1.05 | 0.81-1.36 |
| **Statin users >60 yrs at diagnosis** |  |  |  |  |
| statin use before melanoma diagnosis (*N*=89)^a^ | 284 | 24 | 1.01 | 0.63-1.62 |
| statin use after melanoma diagnosis (*N*=127)^b^ | 420 | 29 | 0.82 | 0.51-1.32 |
| per additional year of use | 420 | 29 | 0.93 | 0.79-1.10 |

Abbreviations

CI, confidence interval; HR, hazard ratio.

^a^Min. 2 dispensings for statin within 1 year prior to melanoma diagnosis and use at time of melanoma diagnosis. Time-fixed analysis.

^b^Statin user since first dispensing of statin after melanoma diagnosis. Time-dependent analysis.

^c^Adjusted for age, sex, tumour thickness and nodal status.

**Table S4- Supplemental table (online only)**

Cox proportional hazard ratios (HR) for all-cause mortality for duration and dose of statin use after melanoma diagnosis.

|  | | **Person years** | **Events** | **Multivariate HR^a^** | **95% CI** | |
| --- | --- | --- | --- | --- | --- | --- |
|  |  |  |  |  | **lower** | **upper** |
| **statin users after melanoma diagnosis, duration of exposure in categories** | **n.a.** |  |  |  |  |  |
| 0 |  | 2077 | 118 | 1 |  |  |
| 0-3 years |  | 402 | 31 | 0.81 | 0.54 | 1.21 |
| >3 years |  | 154 | 10 | 0.98 | 0.48 | 2.00 |
| **statin users after melanoma diagnosis, per 1 DDD** | **Non-user** | 2077 | 118 | 1 |  |  |
|  | **User** | 555 | 41 | 0.97 | 0.71 | 1.33 |
| Male |  | 319 | 24 | 0.72 | 0.47 | 1.12 |
| Female |  | 236 | 17 | 1.64 | 1.00 | 2.69 |
| **statin users after melanoma diagnosis, per DDD category** | **n.a.** |  |  |  |  |  |
| 0 |  | 2077 | 118 | 1 |  |  |
| <1 DDD |  | 346 | 20 | 0.70 | 0.44 | 1.14 |
| >=1 DDD |  | 209 | 21 | 1.04 | 0.65 | 1.68 |
| **statin users after melanoma diagnosis, combined catgories of exposure and average DDD** | **n.a.** |  |  |  |  |  |
| No STAT (0) |  | 2077 | 118 | 1 |  |  |
| <2 yrs and <1 DDD |  | 188 | 11 | 0.61 | 0.33 | 1.15 |
| <2 yrs and >=1 DDD |  | 124 | 14 | 1.07 | 0.61 | 1.89 |
| >=2 yrs and <1 DDD |  | 158 | 9 | 0.86 | 0.42 | 1.74 |
| >=2 yrs and >=1 DDD |  | 85 | 7 | 0.99 | 0.44 | 2.21 |
| **male** |  |  |  |  |  |  |
| No STAT (0) |  | 996 | 81 | 1 |  |  |
| <2 yrs and <1 DDD |  | 99 | 7 | 0.57 | 0.26 | 1.24 |
| <2 yrs and >=1 DDD |  | 76 | 8 | 0.83 | 0.39 | 1.74 |
| >=2 yrs and <1 DDD |  | 85 | 6 | 0.93 | 0.39 | 2.23 |
| >=2 yrs and >=1 DDD |  | 58 | 3 | 0.51 | 0.15 | 1.67 |
| **female** |  |  |  |  |  |  |
| No STAT (0) |  | 1081 | 37 | 1 |  |  |
| <2 yrs and <1 DDD |  | 89 | 4 | 0.69 | 0.24 | 1.99 |
| <2 yrs and >=1 DDD |  | 48 | 6 | 1.43 | 0.59 | 3.43 |
| >=2 yrs and <1 DDD |  | 72 | 3 | 0.74 | 0.22 | 2.56 |
| >=2 yrs and >=1 DDD |  | 27 | 4 | 3.99 | 1.27 | 12.53 |

Abbreviations

CI, confidence interval; DDD, defined daily dose; HR, hazard ratio; n.a., not applicable.

^a^Adjusted for age, sex, tumour thickness and nodal status.
